# Rat model of cancer-induced bone pain: changes in nonnociceptive sensory neurons in vivo

**DOI:** 10.1097/PR9.0000000000000603

**Published:** 2017-06-22

**Authors:** Yong Fang Zhu, Robert Ungard, Natalie Zacal, Jan D. Huizinga, James L. Henry, Gurmit Singh

**Affiliations:** a Michael G. DeGroote Institute for Pain Research and Care, McMaster University, Hamilton, ON, Canada; b Department of Pathology & Molecular Medicine, McMaster University, Hamilton, ON, Canada; c Department of Medicine, Farncombe Family Digestive Health Research Institute, McMaster University, Hamilton, ON, Canada; d Department of Psychiatry and Behavioural Neurosciences, McMaster University, Hamilton, ON, Canada

**Keywords:** Cancer-induced bone pain, Electrophysiology, Sensory neurons, Rat model

## Abstract

**Introduction:**

Clinical data on cancer-induced bone pain (CIBP) suggest extensive changes in sensory function. In a previous investigation of an animal model of CIBP, we have observed that changes in intrinsic membrane properties and excitability of dorsal root ganglion (DRG) nociceptive neurons correspond to mechanical allodynia and hyperalgesia.

**Objectives:**

To investigate the mechanisms underlying changes in nonnociceptive sensory neurons in this model, we have compared the electrophysiological properties of primary nonnociceptive sensory neurons at <1 and >2 weeks after CIBP model induction with properties in sham control animals.

**Methods:**

Copenhagen rats were injected with 10^6^ MAT-LyLu rat prostate cancer cells into the distal femur epiphysis to generate a model of CIBP. After von Frey tactile measurement of mechanical withdrawal thresholds, the animals were prepared for acute electrophysiological recordings of mechanically sensitive neurons in the DRG in vivo.

**Results:**

The mechanical withdrawal threshold progressively decreased in CIBP model rats. At <1 week after model induction, there were no changes observed in nonnociceptive Aβ-fiber DRG neurons between CIBP model rats and sham rats. However, at >2 weeks, the Aβ-fiber low-threshold mechanoreceptors (LTMs) in CIBP model rats exhibited a slowing of the dynamics of action potential (AP) genesis, including wider AP duration and lower AP amplitude compared with sham rats. Furthermore, enhanced excitability of Aβ-fiber LTM neurons was observed as an excitatory discharge in response to intracellular injection of depolarizing current into the soma.

**Conclusion:**

After induction of the CIBP model, Aβ-fiber LTMs at >2 weeks but not <1 week had undergone changes in electrophysiological properties. Importantly, changes observed are consistent with observations in models of peripheral neuropathy. Thus, Aβ-fiber nonnociceptive primary sensory neurons might be involved in the peripheral sensitization and tumor-induced tactile hypersensitivity in CIBP.

## Abstract 2

Nonnociceptive sensory neurons relate to transient episodes of intense pain that characterize neuropathic pain. They are involved in the peripheral sensitization and tactile hypersensitivity.

## 1. Introduction

Cancer-induced bone pain (CIBP) is often severe and intractable and is a significant contributing factor determining morbidity and quality of life.^6,18,22,23,27^ It is induced by many processes including pathological remodeling of the bone and nervous system and characterized by cellular, tissue, and systemic changes that occur during cancer proliferation, invasion, and metastasis.^7,29,30,35^

Although the etiology of cancer pain remains unclear, animal models of CIBP have unraveled neuropathologic processes that occur in the region of tumor growth. Tumour growth can directly induce structural damage of surrounding tissue including sensory neurons in bone. In addition, cancer cells and associated stromal and immune cells secrete factors that can directly activate sensory fibers. These factors lead to a unique pain state that includes aspects of nociceptive, neuropathic, and inflammatory pain.^7,8^ Previous studies have described changes in the functional properties of neurons in the dorsal root ganglion (DRG) and in the dorsal horn of the spinal cord.^4,7,16,31,34–36^ To understand the pathology of the complex state of CIBP, it is essential to determine how these functional changes are associated with different stages of development of CIBP.

It has been suggested that inflammatory pain models are associated with changes only in small DRG neurons, possibly C- and Aδ-fiber nociceptive neurons. However, changes in large nonnociceptive Aβ-fiber neurons are also observed in neuropathic models. In a mouse model of cancer pain, Cain et al.^4^ showed that tumor growth produces physiological and morphological alterations in primary afferent fibers that are characterized by spontaneous activity and sensitization of C-fiber nociceptors. They also recorded lower response thresholds of Aβ-fiber low-threshold mechanoreceptor (LTM) neurons in vivo.^4^ We have reported that in a rat model of CIBP C-, Aδ-, and Aβ-fiber, nociceptive neurons undergo changes in excitability and functional properties and thus might play a role in CIBP.^38^

Therefore, the aim of the current study was to characterize the electrophysiological properties of Aβ-fiber LTMs, comparing these properties in sham control animals and in CIBP animals at <1 and >2 weeks after model induction. We report here that Aβ-fiber LTM neurons at >2 weeks show differences in action potential (AP) configuration and excitability. The patterns of these changes are consistent with observations in animal models of peripheral neuropathy and thus might play a role in the tactile hypersensitivity in CIBP.

## 2. Methods

### 2.1. Experimental rats and tumor induction

All experimental procedures were in accordance with the Care and Use of Laboratory Animals as edited by Canadian Council on Animal Care, and all protocols were reviewed and approved by the McMaster University Animal Research Ethics Board. Male Copenhagen rats (Harlan Laboratories Inc, Indianapolis, IN) weighing 200 to 250 g were randomly assigned to groups and induced as a model of CIBP as described in our previous study.^9,38^ Briefly, CIBP rats were anesthetised with inhaled isoflurane (1%–5% in oxygen) and 5.0 × 10^6^ MAT-LyLu (MLL) cells suspended in 0.05-mL phosphate-buffered saline (PBS) were injected into the distal epiphysis of the femur by manual rotation of a 25-gauge needle between the medial and lateral condyles. Sham injection (control) rats received an injection of 0.05-mL PBS only by the same procedures. Volume of injected material was minimized to ensure that it remained within the penetrated epiphysis, and surgical procedures were minimized to reduce the confounding influence of pain resulting from bone and soft tissue damage.

### 2.2. von Frey test of paw withdrawal threshold

In all cases, behavioral tests were performed immediately before anesthesia for electrophysiological recordings to quantify the development of tactile hypersensitivity characteristic of CIBP. Rats were placed in a transparent Plexiglas box containing 0.5-cm diameter holes spaced 1.5 cm apart on the floor to allow full access to the paws.^24–26,37–39^ Animals were allowed to habituate to the box until cage exploration and major grooming activities had ceased.

von Frey filaments (Stoelting Co, Wood Dale, IL) were applied to the plantar surface of the ipsilateral hind paw to determine mechanical withdrawal thresholds using the up-down method of Dixon.^11^ A von Frey filament was applied 5 times for 3 to 4 seconds each at 3-second intervals to a different spot on the plantar surface of the ipsilateral hind paw. Filaments were applied in ascending order of force until a clear withdrawal response was observed. When this occurred, the next lightest filament was reapplied, and the process continued until a 50% withdrawal response threshold was derived.^5^ Brisk foot withdrawal in response to the mechanical stimulus was interpreted as indicating mechanical hypersensitivity.

### 2.3. Intracellular recording in vivo

Details of acute intracellular electrophysiological recording techniques have been reported previously in animal models of neuropathic pain.^32,37–39^ In brief, each rat was initially anesthetised with a mixture of ketamine, xylazine, and acepromazine delivered intraperitoneally. The right jugular vein was catheterized for intravenous infusion of drugs and the rat was fixed in a stereotaxic frame and the vertebral column rigidly clamped at the L2 and L6 vertebral levels. The right femur was fixed by a customized clamp onto the stereotaxic frame to minimize movement of the DRG during mechanical searching for receptive fields on the leg. The L4 DRG was selected for study, as it contains large numbers of hind leg afferent somata. A laminectomy was performed to expose the ipsilateral L4 DRG. The L4 dorsal root was sectioned close to the spinal cord and placed on a bipolar electrode (FHC, Bowdoinham, ME) used for electrical stimulation. The exposed spinal cord and DRG were covered with warm paraffin oil at 37°C to prevent drying. Rectal temperature was maintained at 37°C using a temperature-controlled infrared heating lamp.

For recording, each rat was maintained at a surgical level of anesthesia using sodium pentobarbital (20 mg/kg; Ceva Sante Animal, Libourne, France) and was mechanically ventilated through a tracheal cannula using a Harvard Ventilator (Model 683; Harvard Apparatus, QC, Canada). The ventilation parameters were adjusted so that end-tidal CO_2_ concentration was maintained around 40- to 50-mm Hg, as measured using a CapStar-100 End-Tidal CO_2_ analyzer (CWE, Ardmore, PA). Immediately before the start of recording, an initial 1-mg/kg dose of pancuronium (Sandoz, Boucherville, QC, Canada) was given to eliminate muscle tone. The effects of pancuronium were allowed to wear off periodically to confirm a surgical level of anesthesia; this was monitored by observing pupil diameter and response to noxious pinch of a forepaw. Supplementation of pentobarbital and pancuronium was administered at doses of 1/3 of the previous dose, approximately each hour through the jugular catheters.

Intracellular recordings from somata in the exposed DRG were made with borosilicate glass micropipettes (1.2 mm outside diameter, 0.68 mm inside diameter; Harvard Apparatus, Holliston MA). The electrodes were pulled using a Brown-Flaming pipette puller (model P-87; Sutter Instrument Co, Novota, CA). These electrodes were filled with 3M KCl (DC resistance 50–70 MΩ). Signals were recorded with a MultiClamp 700B amplifier (Molecular Devices, Union City CA) and digitized on-line through Digidata 1322A interface (Molecular Devices) with pCLAMP 9.2 software (Molecular Devices). The microelectrode was advanced using an EXFO IW-800 micromanipulator (EXFO, Montreal, QC, Canada) in 2-μm steps until an abrupt hyperpolarization of at least 40 mV appeared. Once a stable membrane potential had been confirmed, a single stimulus was applied to the dorsal root to provoke an AP. The protocol editor function in the pCLAMP 9.2 software was used to evoke a somatic AP by stimulation with a single rectangular intracellular depolarizing voltage pulse.

### 2.4. Action potential configuration

As described in our pervious article,^38,39^ the first AP evoked by stimulation of the dorsal root and measured at the DRG soma in each neuron was used to compare the configuration between control and cancer rats. Criteria for acceptance of neurons in the subsequent analysis included a stable resting membrane potential (Vm) more negative than −40 mV with a somatic spike evoked by dorsal root stimulation that was >40 mV. Variables in AP configuration included Vm, AP amplitude (APA), AP duration at base (APdB), AP rise time (APRT), AP fall time (APFT), afterhyperpolarization amplitude (AHPA), and afterhyperpolarization duration to 50% recovery (AHP50).

### 2.5. Conduction velocity

The distance from the stimulation site (cathode) to the recording site (center of the DRG) was measured at the end of the experiment to determine the conduction distance.^39^ This value was used to calculate the conduction velocity (CV) of the dorsal root axon associated with each neuron.

### 2.6. Dorsal root ganglion neuron classification

Recorded neurons were classified as C-, Aδ-, or Aβ-fibers neurons based on their CV, AP configuration, and their receptive properties defined using hand-held mechanical stimulators.^12,13,37–39^ The differentiation of high-threshold mechanoreceptor neurons vs LTM neurons was based on their sensory properties identified during receptive field searching. High-threshold mechanoreceptor neurons responded to noxious stimuli including noxious pressure, pinch, and probing with fine forceps, a sharp needle, coarse-toothed forceps, or coarse flat forceps, whereas LTM neurons responded to innocuous stimuli such as a moving brush, light pressure with a blunt object, light manual tap, or vibration. Besides the threshold of activation, the rate of adaption and the tissue location of the receptive field were other major factors used to further classify Aβ-fiber LTM neurons as guard or field hair (GF) neurons, glabrous skin neurons, Pacinian neurons, slowly adapting (SA) neurons, and muscle spindle (MS) neurons. GF neurons were rapidly adapting (RA) cutaneous neurons. Glabrous and Pacinian neurons were both RA nonhair neurons and were named RA neurons. Slowly adapting neurons were SA cutaneous neurons. Muscle spindle neurons were SA neurons with deep subcutaneous receptive fields activated by deep tissue manipulation of the muscle belly but not by cutaneous stimulation.

### 2.7. Excitability of soma

To quantify soma excitability, the threshold of depolarizing current pulses injected into the soma was performed by applying pulses of 20 milliseconds in increments of 0.05 nA through the recording electrode until an AP was elicited or until a maximum current of 4 nA was reached.^37,38^ The excitability of the soma was also evaluated by comparing the number of APs evoked by injecting defined current pulses to the DRG soma; 3 intracellular current injections of 100 milliseconds each were delivered with 1 and 2 nA.

### 2.8. Excitability of the receptive field measured by responses to application of von Frey filaments

To determine whether changes in properties of peripheral receptors might contribute to the mechanical hypersensitivity that characterizes this model, von Frey filaments were applied to the peripheral receptive fields of neurons studied in the electrophysiological recordings. Calibrated von Frey filaments were applied to the identified receptive field areas as a tactile stimulation, and the minimum filament that elicited an AP in the soma was recorded.^37,38^

### 2.9. X-ray radiographs

After electrophysiological recordings, animals were killed without recovery by anesthetic overdose. Ipsilateral hind limbs of all rats were immediately dissected, shed of most cutaneous tissue and muscle, and immediately fixed in a 10% formalin solution in PBS. High-resolution radiographic scans of dissected rat femurs were then taken with a Faxitron X-ray MX-20 system (Faxitron, Wheeling, IL) on Kodak MIN-R 2000 Mammography Film (Eastman Kodak, Rochester, NY).^9,38^

### 2.10. Statistical analysis

Data are presented as mean ± the SEM. Response data were analyzed with Mann–Whitney *U* tests. *P* < 0.05 was considered to indicate a significant difference as shown in the graphs. GraphPad Prism software (GraphPad Software, Inc, La Jolla, CA) was used for all statistical analyses and graphing.^37–39^

## 3. Results

### 3.1. Behavioral evidence

**Figure 1.**
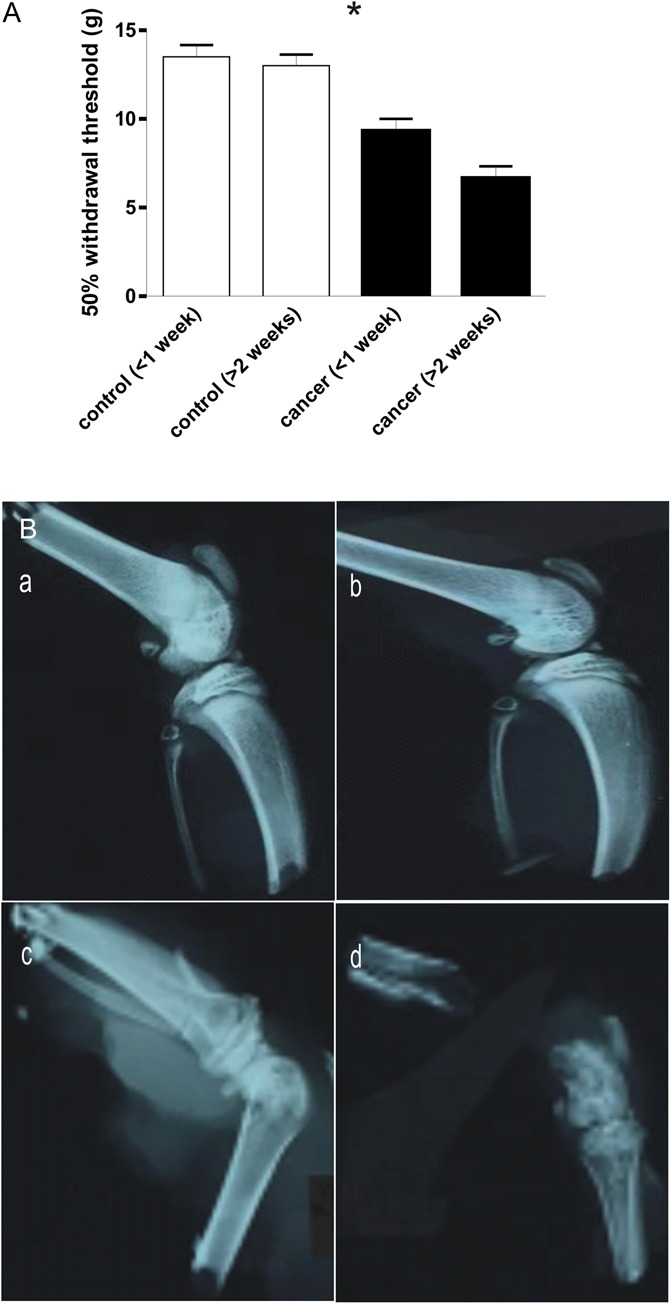
Bone tumors induce structural changes and nociception. (A) Comparison of 50% withdrawal threshold between control and cancer rats. Withdrawal threshold in response to mechanical stimulation of the plantar surface of the ipsilateral hind paw with von Frey filaments was recorded on the same day immediately before the acute electrophysiological experiment in control (<1 week, n = 6; >2 weeks, n = 6) and cancer (<1 week, n = 6; >2 weeks, n = 6) animals. The significant differences between each group animals are shown in Table 1. (B) Representative radiographs of rat ipsilateral hind legs. Radiographs of the ipsilateral hind leg from control (a, 6 days; c, 18 days) and MLL cell–injected (b, 6 days; d, 18 days) rats displaying structural changes following model induction. *P < 0.05. The absence of an asterisk indicates the lack of a statistically significant difference. Mann–Whitney *U* test.

Withdrawal thresholds of the ipsilateral hind legs of the sham PBS (control) and MLL cell–injected rats (CIBP) were compared. After injection, CIBP rats developed behavioral evidence suggesting pain perception in this limb. Behavioral tests of tactile hypersensitivity were performed measuring paw withdrawal threshold from von Frey filaments (Fig. 1A). Stimulation of the plantar surface of the hind paw evoked a withdrawal response in control rats at pressures only above 10 g at all time points. Filaments to which the control rats showed no withdrawal response, ie, 4.0 to 8.0 g, however, evoked a clear withdrawal of the tumour-bearing hind limb in CIBP rats. Furthermore, the withdrawal threshold response decreased further with increasing days in CIBP model rats. Withdrawal thresholds were 13.50 ± 1.64 g in control rats (n = 6, <1 week) and 9.33 ± 1.63 g in CIBP rats (n = 6, <1 week); *P* = 0.004, and were 13.00 ± 1.55 g in control rats (n = 6, >2 weeks) and 6.67 ± 1.63 g in CIBP rats (n = 6, >2 weeks), *P* = 0.002. Table 1 shows all the comparison *P* values between every 2 groups from 4 groups (control and CIBP rats at <1 and >2 weeks, each). Evidence for different levels of osteolytic degradation was also visible in the radiographs of MLL cell–injected ipsilateral hind limbs. Representative radiographs are illustrated in Figure 1B.

**Table 1:**
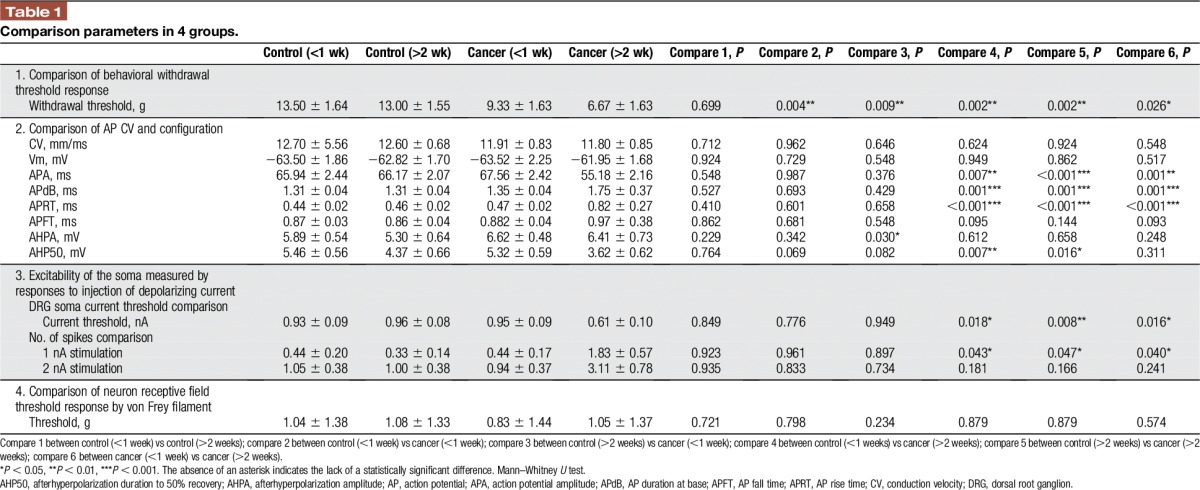
Comparison parameters in 4 groups.

### 3.2. Electrophysiology measurement

All neurons included in this study were Aβ nonnociceptive neurons judged by sensory testing and by AP features. Electrophysiological properties of Aβ-fiber LTMs in control rats were comparable with those of the CIBP rats at <1 week and >2 weeks. A total of 18 neurons from 6 animals for each group met the acceptance criteria. In terms of the breakdown of different types of Aβ-fiber LTMs, both groups of animals yielded comparable numbers of each neuronal subtype following the criteria of Lawson et al (1997). Aβ-fiber LTMs were included based on the 4 subsets: 4 guard/field hair, 4 RA, 4 SA, and 6 MS in each group.

### 3.3. Action potential conduction velocity and configuration

**Figure 2.**
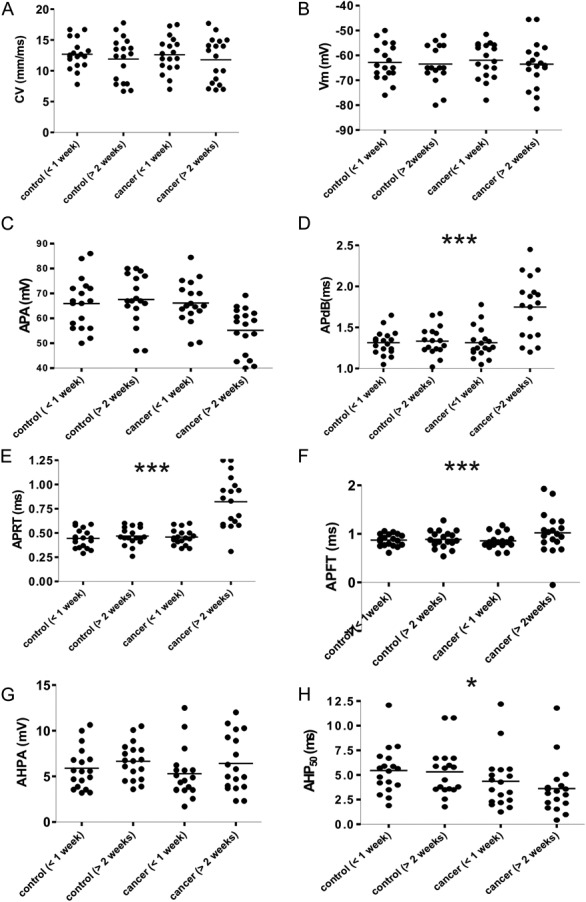
Changes of action potential configuration of Aβ-fiber low-threshold mechanoreceptor neurons in control and cancer rats. Scatter plots show the distribution of the variables with the median (horizontal line) superimposed in nonnociceptive Aβ-fiber low-threshold mechanoreceptor neurons. Panels are as follows: (A) conduction velocity (CV); (B) resting membrane potential (Vm); (C) action potential (AP) amplitude (APA); (D) AP duration at base (APdB); (E) AP rise time (APRT); (F) AP fall time (APFT); (G) afterhyperpolarization amplitude below Vm (AHPA); and (H) afterhyperpolarization duration to 50% recovery (AHP50). The significant differences between each group animals are shown in Table 1. An asterisk in the figure indicates the significant differences between cancer (<1 week) and cancer (>2 weeks). *P < 0.05, ***P < 0.001. The absence of an asterisk indicates the lack of a statistically significant difference. Mann–Whitney *U* test.

Intracellular somatic APs evoked by electrical stimulation of the dorsal root showing the electrophysiological parameters were measured, including: (1) CV; (2) Vm; (3) APdB; (4) APRT; (5) APFT; (6) APA; (7) AHP50%; and (8) AHPA. All data are shown in the scatter plots of Figure 2, illustrating the distributions of various parameters for individual neurons in each neuron type in control and CIBP rats. Table 1 shows the statistical comparison between all groups (control and CIBP rats at <1 and >2 weeks, each).

#### 3.3.1. Conduction velocity

Comparison of the neuronal CV between control and CIBP rats at >2 weeks did not show a significant difference. At >2 weeks, the CV was 12.60 ± 0.68 mm/ms in control vs 11.80 ± 0.85 mm/ms in CIBP rats (*P* = 0.924). At <1 week, the CV was 12.70 ± 5.56 mm/ms in control vs 11.91 ± 0.83 mm/ms in CIBP neurons (*P* = 0.962).

#### 3.3.2. Resting membrane potential

Vm of Aβ-fiber LTMs of CIBP rats was not significantly different from Vm of control rats at >2 weeks (control, −62.82 ± 1.70 mV vs CIBP, −63.50 ± 1.86 mV, *P* = 0.862), while also not significantly different at <1 week group (control, −61.95 ± 1.68 mV vs CIBP, −63.52 ± 2.25 mV, *P* = 0.729).

#### 3.3.3. Action potential amplitude

There were significant differences in APA between control and CIBP rats at >2 weeks. There was reduced APA in CIBP rats (control, 66.17 ± 2.07 mV vs CIBP, 55.18 ± 2.16 mV, *P* < 0.001); however, there were no significant differences between control and CIBP rats at <1 week (control, 65.94 ± 2.44 mV vs CIBP, 67.56 ± 2.42 mV, *P* = 0.987).

#### 3.3.4. Action potential duration at base

In marked contrast to neurons in control rats, neurons in CIBP rats exhibited a wider APdB (control, 1.33 ± 0.17 ms vs CIBP, 1.75 ± 0.37 ms; *P* = 0.001) at >2 weeks. No significant differences were observed between groups at <1 week (control, 1.31 ± 0.04 ms vs CIBP, 1.31 ± 0.04 ms; *P* = 0.693).

#### 3.3.5. Action potential rise time

A longer APRT was observed in CIBP rats relative to control at >2 weeks. APRT was 0.46 ± 0.02 milliseconds in control and 0.82 ± 0.27 milliseconds in CIBP rats (*P* < 0.001). At <1 week, there were no differences between groups; APRT was 0.44 ± 0.02 milliseconds in the control rats and 0.47 ± 0.02 milliseconds in the CIBP rats (*P* = 0.601).

#### 3.3.6. Action potential fall time

There were no differences in neuronal APFT between groups at either time point. At >2 weeks, APFT in control animals was 0.89 ± 0.17 milliseconds and 0.97 ± 0.38 milliseconds in CIBP rats (*P* = 0.195). At <1 week, APFT in control animals was 0.87 ± 0.03 milliseconds and 0.88 ± 0.04 milliseconds in the CIBP rats (*P* = 0.275).

#### 3.3.7. AHP amplitude

No differences in AHPA were observed between any groups. At >2 weeks, AHPA in control animals was 5.30 ± 0.64 mv and 6.41 ± 0.73 mv in CIBP rats (*P* = 0.658). At <1 week, there were no differences between groups (control, 5.89 ± 0.54 mv vs CIBP 6.62 ± 0.48 mv, *P* = 0.342).

#### 3.3.8. Afterhyperpolarization duration to 50% recovery

A shorter AHP50 was observed in CIBP rats relative to control at >2 weeks. AHP50 in control animals was 4.37 ± 0.66 ms and 3.62 ± 0.62 ms in CIBP rats, (*P* = 0.016). No differences in AHP50 were observed between groups at <1 week. AHP50 in control animals was 5.46 ± 0.56 ms and 5.32 ± 0.59 ms in CIBP rats (*P* = 0.069).

### 3.4. Changes in action potential configuration in subgroups of Aβ-fiber low-threshold mechanoreceptors

**Figure 3.**
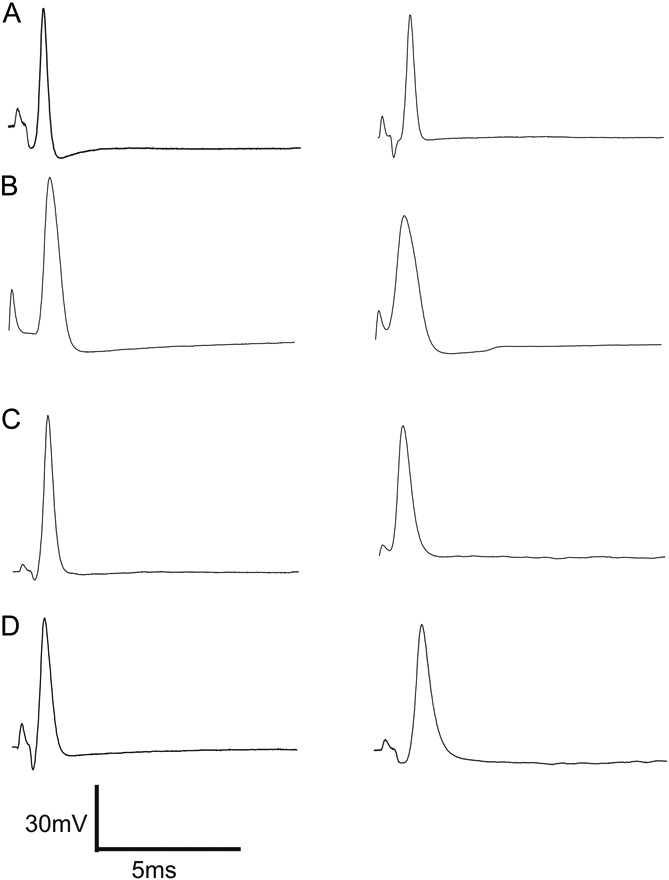
Examples of action potentials recorded from subtypes of Aβ-fiber low-threshold mechanoreceptor neurons. Somatic evoked and recorded intracellularly selected to represent the action potential duration values for each of the different groups of neuron in control (left) and cancer-induced bone pain (right) animals. (A) Muscle spindle neurons; (B) rapidly adapting neurons; (C) slowly adapting neurons; (D) GF neurons.

The Aβ-fiber LTM neurons showed significantly slower dynamics of APA, APdB, and APRT. These parameters were also compared for each subset of Aβ-fiber LTMs based on the 4 subsets described above: GF, RA, SA, and MS neurons. Figure 3 shows representative intracellular somatic APs for these subsets. Muscle spindle neurons were the most affected, followed by RA, SA, and GF neurons.

In MS neurons, the slower dynamics of the AP was the most obvious of these parameters studied. There was a reduced APA in CIBP rats at >2 weeks (control, 56.50 ± 8.02 mV vs CIBP, 46.62 ± 8.02, *P* = 0.04), a longer APdB in CIBP rats at 2 weeks (control, 1.21 ± 0.06 ms vs CIBP, 1.76 ± 0.39 ms; *P* = 0.007), and a longer APRT in CIBP rats at 2 weeks (control, 0.46 ± 0.10 ms vs CIBP, 0.75 ± 0.20 ms, *P* = 0.002).

In RA neurons, there was a reduced APA in CIBP rats at >2 weeks (control, 71.25 ± 4.99 mV vs CIBP, 57.90 ± 8.53, *P* = 0.03) and a longer APdB in CIBP rats at 2 weeks (control, 1.42 ± 0.18 ms vs CIBP, 1.62 ± 0.31 ms; *P* = 0.005) but no significance difference in the ARPT in CIBP rats at 2 weeks (control, 0.51 ± 0.100 ms vs CIBP, 0.87 ± 0.17 ms, *P* = 0.03).

In SA neurons, there was a reduced APA in CIBP rats at >2 weeks (control, 73.25 ± 7.81 mV vs CIBP, 59.06 ± 4.38, *P* = 0.03), a longer APdB in CIBP rats at 2 weeks (control, 1.49 ± 0.13 ms vs CIBP, 1.59 ± 0.25 ms; *P* = 0.11), and a longer APRT in CIBP rats at 2 weeks (control, 0.44 ± 0.13 ms vs CIBP, 0.89 ± 0.37 ms, *P* = 0.11).

In GF neurons, there was no difference in APA in CIBP rats at >2 weeks (control, 74.45 ± 5.31 mV vs CIBP, 61.43 ± 7.06, *P* = 0.06), no difference in APdB in CIBP rats at 2 weeks (control, 1.39 ± 0.11 ms vs CIBP, 1.62 ± 0.27 ms; *P* = 0.11), and no difference in APRT in CIBP rats at 2 weeks (control, 0.45 ± 0.02 ms vs CIBP, 0.81 ± 0.38 ms, *P* = 0.34).

### 3.5. Excitability of neurons

#### 3.5.1. Excitability of the soma measured by responses to injection of depolarizing current

**Figure 4.**
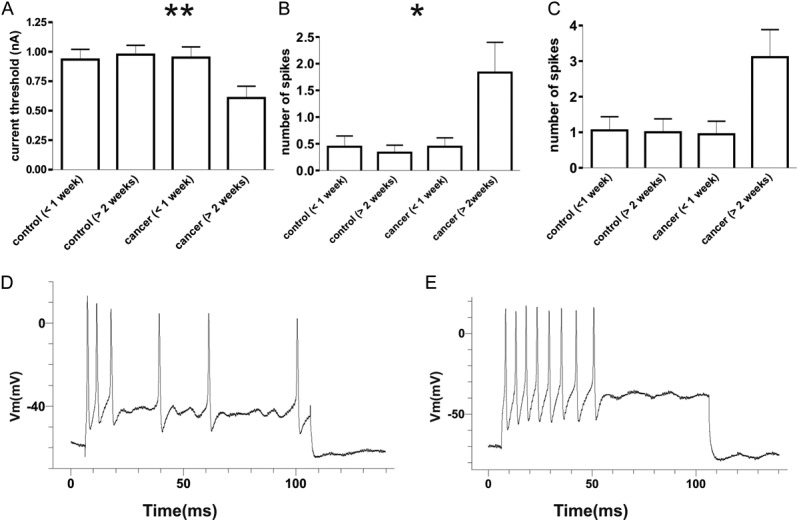
Decreased excitability threshold in nonnociceptive Aβ-fiber low-threshold mechanoreceptor neurons. (A) The current threshold was defined as the minimum current required to evoke an action potential (AP) by intracellular current injection (20 ms). Excitability of the dorsal root ganglion somata was significantly increased at >2 weeks cancer-induced bone pain rats, as indicated by the decreased activation threshold in nonnociceptive Aβ-fiber neurons (A). (B and C) A comparison of the repetitive discharge characteristics of dorsal root ganglion cells produced by intracellular current injection. Columns indicate the number of APs evoked by different magnitudes of intracellular depolarizing current injection in nonnociceptive Aβ-fiber low-threshold mechanoreceptor neurons. (B) 1 nA, 100 milliseconds; (C) 2 nA, 100 milliseconds; (D) Representative examples of raw recordings to demonstrate the greater number of APs evoked by intracellular current injection in muscle spindle neurons at >2 weeks cancer-induced bone pain rat (E) vs at >2 weeks control rats (D). The significant differences between each group animals are shown in Table 1. An asterisk in the figure indicates the significant differences between cancer (<1 week) and cancer (>2 weeks). *P < 0.05, **P < 0.01. The absence of an asterisk indicates the lack of a statistically significant difference. Mann–Whitney *U* test.

The AP responses to intracellular depolarizing current pulse injection were tested to determine whether there is a difference in soma excitability in CIBP model rats. Figure 4A illustrates the threshold currents that elicited APs in different groups of animals. At >2 weeks, the threshold of Aβ-fiber LTM neurons in CIBP rats showed a significant decrease; activation thresholds were 0.61 ± 0.10 nA (n = 18) in CIBP rats and 0.96 ± 0.08 nA (n = 18) in control rats vs (*P* = 0.008). There was no significant difference in Aβ-fiber LTM neurons at <1 week (0.93 ± 0.09 nA in control rats, n = 18 vs, 0.95 ± 0.09 nA in CIBP rats, n = 18; *P* = 0.776).

Figures 4B and C show the number of APs elicited with different current strengths; with a 1 nA, 20-millisecond current injection, the number of APs elicited in control rats at >2 weeks were 0.33 ± 0.14 (n = 18), whereas in CIBP rats, it was 1.83 ± 0.57 (n = 18) (*P* = 0.047). At <1 week, the number of APs in control rats was 0.44 ± 0.20 (n = 18), whereas in CIBP rats, it was 0.44 ± 0.17 (*P* = 0.461) (Fig. 3B). With a 2 nA, 100-millisecond current injection, the number of APs elicited in control rats at >2 weeks were 1.00 ± 0.38 (n = 18), whereas in CIBP rats, it was 3.11 ± 0.78 (n = 18) (*P* = 0.166). At <1 week, the number of APs in control rats was 1.05 ± 0.38 (n = 18), whereas in CIBP rats, it was 0.94 ± 0.37 (*P* = 0.833) (Fig. 3C). Figures 4D and E show typical discharge patterns of APs elicited in MS neurons by 2 nA current pulses with a duration of 100 milliseconds. In this figure, CIBP rats at <1 week showed 6 APs, whereas at >2 weeks, CIBP rats showed 8 APs with the same current pulse injection, which was the maximal number of APs observed using 2 nA current pluses. Table 1 shows all the comparison *P* values between the 4 groups.

### 3.6. Excitability of the receptive field measured by responses to application of von Frey filaments

**Figure 5.**
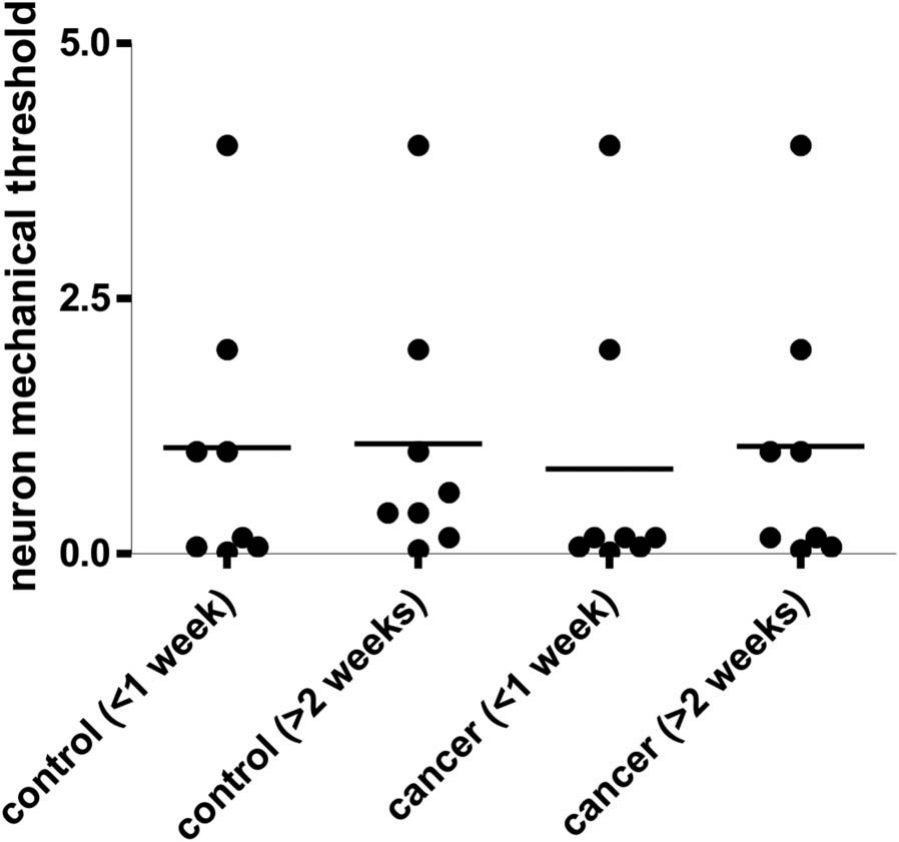
The mechanical threshold in nonnociceptive Aβ-fiber low-threshold mechanoreceptor (LTM) neurons. The mechanical response threshold in nonnociceptive Aβ-fiber LTM neurons was measured by application of von Frey filaments to the peripheral receptive fields. Scatter plots show the threshold distribution of the variables with the median (horizontal line) superimposed in nonnociceptive Aβ-fiber LTM neurons including slowly adapting and rapidly adapting neurons. There is no significant difference in all groups. The differences between each group animals are shown in Table 1. An asterisk in the figure indicates the significant differences between cancer (<1 week) and cancer (>2 weeks). *P < 0.05, **P < 0.01. The absence of an asterisk indicates the lack of a statistically significant difference. Mann–Whitney *U* test.

The mechanical thresholds of DRG neurons tested with von Frey filaments during electrophysiology recording are shown in Figure 5. The mechanical thresholds of RA and SA neurons in control rats and CIBP rats were within the range 0.07 to 4 g and 0.02 to 4 g, respectively. At >2 weeks, the threshold of these LTM neurons in CIBP rats showed no significant difference; activation thresholds were 1.05 ± 1.37 g (n = 8) in CIBP rats vs 1.08 ± 1.33 g (n = 8) in control rats (*P* = 0.875). There was no significant difference in Aβ-fiber LTM neurons at <1 week (1.04 ± 1.38 g, (n = 8) in control rats vs 0.83 ± 1.44 g (n = 8) in CIBP rats (*P* = 0.798)). Table 1 shows the comparison *P* values between the 4 groups.

## 4. Discussion

In our behavior studies, the mechanical withdrawal threshold response decreased with increasing duration of the CIBP animal model. Multiple mechanisms could account for these changes in nociception, including lowered activation threshold of nociceptive small Aδ-fiber neurons and C-fiber neurons. Studies from our laboratory and others have also suggested a possible role of Aβ-fiber LTMs in nociceptive mechanisms, such as allodynia and mechanical hypersensitivity.^1,17,20,28,37,39^ One possible explanation is that some Aβ-fiber LTM neurons may take up a new functional role in nociception and begin to convey signals along novel pathways leading to nociception during CIBP model development. We found that after 2 weeks, Aβ-fiber LTM neurons in CIBP model animals show differences in AP configurations and excitability, similar to what has been reported in an animal model of peripheral neuropathy.^37,39^ The correlation of the changes between the function of Aβ-fiber LTM neurons and behavioral nociception suggests the potential participation of Aβ-fiber LTM neurons in bone cancer pain generated in the present model.

Previous studies have indicated that the CIBP state includes aspects of nociceptive, neuropathic, and inflammatory pain.^7,8^ This prompted us to further question the role that Aβ-fiber LTM neurons fulfil in CIBP. In various animal models of chronic pain, there is evidence that inflammation and neuropathic etiologies affect distinct populations of DRG neurons. In peripheral models of inflammatory pain induced by injecting complete Freund adjuvant subcutaneously, only small Aδ-fiber neurons and C-fiber neurons undergo significant changes in electrophysiological properties.^33^ In hind leg joint inflammation models, indirect evidence suggests that large, nonnociceptive A-fiber neurons are unaffected.^3,15^

On the contrary, in peripheral neuropathic pain models, changes in large Aβ-fiber LTM neurons are commonly reported, such as in the complete sciatic nerve transection model,^2^ the partial sciatic nerve transection model,^19^ and the sciatic nerve cuff model.^37,39^ Although in some studies on neuropathic models changes in C-fiber neurons have been reported,^1,17,21^ such changes are less prominent than those in A-fiber neurons. Therefore, we propose that the electrophysiological changes in Aβ-fiber LTM neurons may be associated with a neuropathic etiology that follows model induction of animal models of CIBP. In fact, the observed changes in AP configuration in Aβ-fiber LTM neurons, including wider AP duration, and lower APA, reflect slowed dynamics of depolarization that are consistent with observations in models of peripheral neuropathy.^37,39^

It is not clear what is driving the changes in Aβ-fiber LTM neurons or how these neurons are affected. A possible explanation is that tumor growth induces peripheral nerve lesions on sensory neurons. Tumor cells invade the normal tissue, come into contact, compress, and injure the processes of sensory neurons including Aβ-fiber LTM neurons; Cain et al showed degeneration of nerve fibers in the skin in their murine model of cancer pain^4^ This implies that a component of CIBP is of neuropathic origin. A slowing of the dynamics of AP configuration in these neurons suggests a change in sodium currents in these neurons, either a functional change or a change in expression of channels.^10,14^ However, the specific ionic mechanisms remain unknown.

We did not find a change in the threshold of activation of the peripheral receptive field of these neurons as measured by responses to application of von Frey filaments in the CIBP rats. This is an important observation in view of earlier suggestion that CIBP is at least partially a neuropathic pain, which is characterized by tactile hypersensitivity. Specifically, we have reported that in a rat model of prostate CIBP, all 3 types of primary sensory neurons undergo increases in excitability corresponding to increases in CIBP behaviors.^37^

Given that behavioral reflex studies demonstrated a decrease in mechanical withdrawal threshold but the Aβ-fiber LTM neurons did not show a change in activation threshold of the peripheral receptive fields, we interpret these data to suggest that the behavioral change may be due to increased ectopic activity in Aβ-fiber LTM neurons, increased excitability of the soma of Aβ-fiber LTM neurons in a CIBP model as reported in a model of peripheral neuropathic pain^37,39^

## 5. Conclusion

Results from this study support the concept that nonnociceptive Aβ LTM neurons undergo changes in the model of CIBP. Importantly, there is a delayed onset of electrophysiological changes in these neurons, corresponding with changes in nociceptive behavioral scoring. The time course of development of the phenotypic changes in sensory neurons in these models may relate to the transient episodes of intense pain that characterize CIBP and the changes specifically in Aβ low-threshold LTM neurons might account for the relatively refractory nature of this type of pain, especially in more advanced stages.

## Disclosures

The authors have no conflicts of interest to declare.

This study was supported by the Canadian Institutes of Health Research, Prostate Cancer Canada, and a postdoctoral fellowship for YFZ from the Michael G. DeGroote Institute for Pain Research and Care.
